# Microbial community response reveals underlying mechanism of industrial-scale manganese sand biofilters used for the simultaneous removal of iron, manganese and ammonia from groundwater

**DOI:** 10.1186/s13568-017-0534-7

**Published:** 2018-01-08

**Authors:** Yu Zhang, Rui Sun, Aijuan Zhou, Jiaguang Zhang, Yunbo Luan, Jianna Jia, Xiuping Yue, Jie Zhang

**Affiliations:** 1 State Key Laboratory of Urban Water Resource and Environment, Harbin Institute of Technology, Harbin, 150090 China; 2 College of Environmental Science and Engineering, Taiyuan University of Technology, Taiyuan, 030024 China; 3 State Key Laboratory Breeding Base of Coal Science and Technology Co-founded by Shanxi Province and the Ministry of Science and Technology, Taiyuan University of Technology, Taiyuan, 030024 China; 4 College of Architecture and Civil Engineering, Taiyuan University of Technology, Taiyuan, 030024 China; 5 College of Mechanics, Taiyuan University of Technology, Taiyuan, China; 6 Beijing Enterprises Water Group Limited, Beijing, 100195 China

**Keywords:** Groundwater, Iron, manganese and ammonia removal, Industrial-scale biofilters, Microbial community, Canonical correspondence analysis (CCA)

## Abstract

Most studies have employed aeration–biofiltration process for the simultaneous removal of iron, manganese and ammonia in groundwater. However, what’s inside the “black box”, i.e., the potential contribution of functional microorganisms behavior and interactions have seldom been investigated. Moreover, little attention has been paid to the correlations between environmental variables and functional microorganisms. In this study, the performance of industrial-scale biofilters for the contaminated groundwater treatment was studied. The effluent were all far below the permitted concentration level in the current drinking water standard. Pyrosequencing illustrated that shifts in microbial community structure were observed in the microbial samples from different depths of filter. Microbial networks showed that the microbial community structure in the middle- and deep-layer samples was similar, in which a wide range of manganese-oxidizing bacteria was identified. By contrast, canonical correlation analysis showed that the bacteria capable of ammonia-oxidizing and nitrification was enriched in the upper-layer, i.e., *Propionibacterium*, *Nitrosomonas*, *Nitrosomonas* and *Candidatus Nitrotoga*. The stable biofilm on the biofilter media, created by certain microorganisms from the groundwater microflora, played a crucial role in the simultaneous removal of the three pollutants.

**Electronic supplementary material:**

The online version of this article (10.1186/s13568-017-0534-7) contains supplementary material, which is available to authorized users.

## Introduction

Groundwater is an important source for public water systems in some districts, especially in northeast and north-central China (Tang et al. 2016). However, most groundwater contains a variety of inorganic chemicals, such as iron and manganese, which may give water an unpleasant metallic taste, staining of laundry and even causing diseases in humans (e.g., neurotoxicity caused by manganese). Moreover, ammonia, usually coexisting with iron and manganese, needs to be removed before water disinfection process, because it could react with chlorine and produce carcinogens (chloramines) (Tekerlekopoulou et al. 2013). What’s more, the chemical reaction between ammonia and manganese could form precipitates, resulting in muddying the water and further clogging pipes (Hasan et al. 2013). Thus, to avoid these inorganic contaminants introducing into water supplies, it is crucial to simultaneously remove iron, manganese and ammonia from groundwater.

**Fig. 1:**
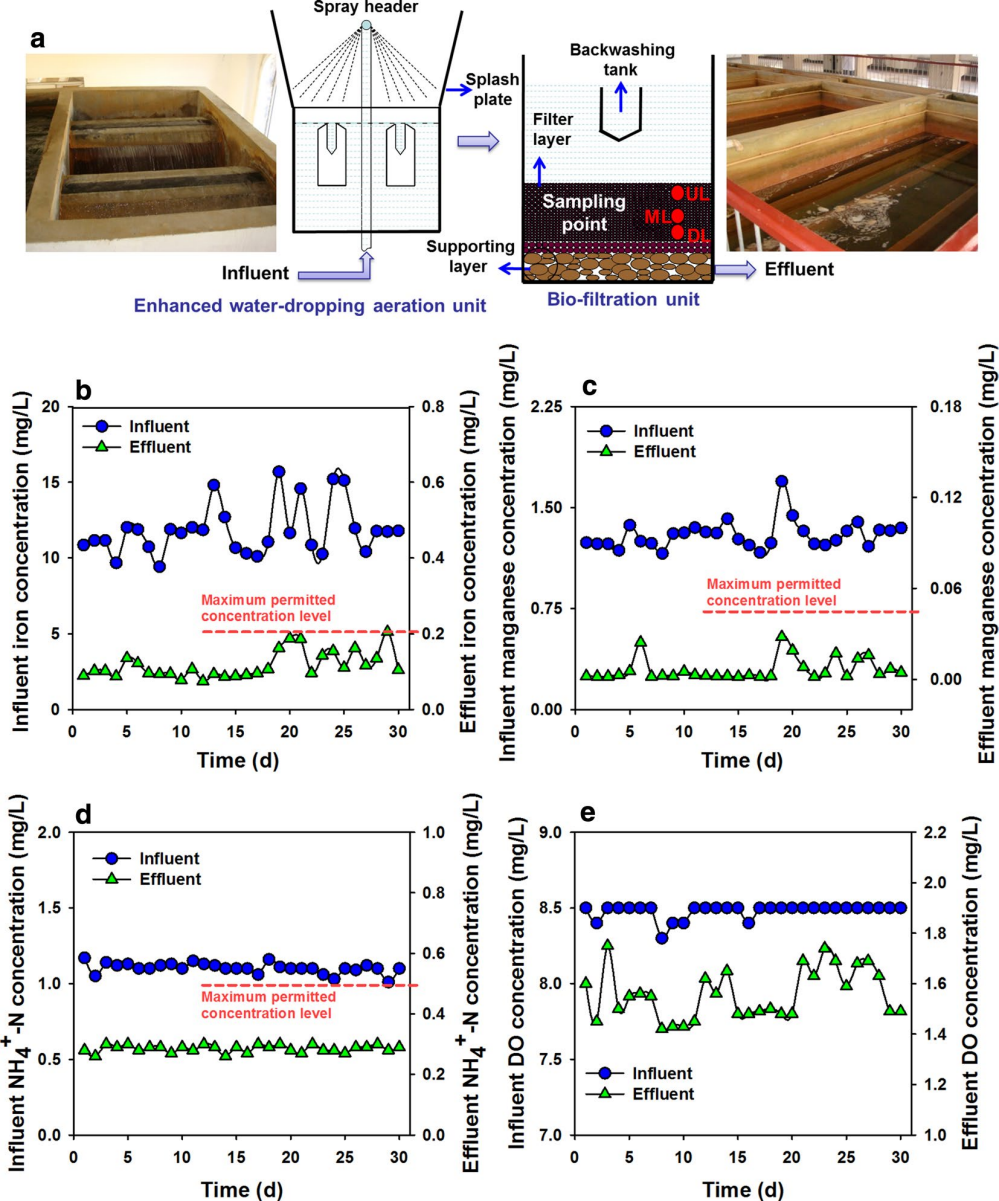
Schematic and photograph of the enhanced water-dropping aeration and biofiltration process (**a**). Iron (**b**), manganese (**c**) and ammonia (**d**) removal effects and the DO variation (**e**) in the enhanced system

To date, various approaches, involving physical, chemical or biological technologies, have been developed to enhance potable water quality by removing these inorganic contaminants (Tekerlekopoulou et al. 2013). Recently, the simultaneous biological removal of the above pollutants by one or more biofiltration or combined pretreatment have emerged (Pacini et al. 2005; Tekerlekopoulou et al. 2013). These methods were characterized of cost-effective, non-additional chemicals and high treatment efficiency. Pre-treatment with aeration before biofiltration have been concluded to be a promising approach for increasing the treatment capacity, simplifying the treatment process and reducing investment costs (Li et al. 2013; Štembal et al. 2005). In northeast China, the Songbei Water Treatment Plant (WTP) in Harbin City (China) employs the aeration–biofiltration process (Fig. 1a) to obtain the simultaneous removal of the three pollutants. Our previous study has shown that high removal efficiencies of iron, manganese and ammonia achieved up to 98, 95 and 80%, respectively, when high dissolved iron (~ 15 mg/L), manganese (~ 1.5 mg/L) and ammonia (~ 1.0 mg/L) exist in the groundwater (Li et al. 2013). Although much is known about the aeration–biofiltration process contributing to the performance of iron, manganese and ammonia removal, what’s inside the “black box”, i.e., the potential contribution of functional microorganisms behavior and interactions have seldom been investigated. Additional exploration of the microbial communities will allow engineers and researchers to establish more direct cause-and-effect relationships between community structure and function.

Based on the above-mentioned considerations, we monitored the dissolved iron, manganese, ammonia and oxygen (DO) concentrations during 30 days stable operation of the Songbei WTP. The microstructure of mature biofilter media was also analyzed by scanning electron microscope (SEM) and energy disperse spectroscopy (EDS). We also examined the related functional microbial community structures, using high-throughput pyrosequencing of the 16S rRNA gene. Microbial networks were visualized in Cytoscape v3.2.1 for depicting the similarity and difference between the different microbial consortia from different depths of filter layer. Correlations between environmental variables (iron, manganese, ammonia, DO, temperature and layer height) and functional microorganisms (16 characteristic bacteria) were assessed, using canonical correspondence analysis (CCA).

## Materials and methods

### Description of sampling sites

The study was performed on the Songbei WTP, which was put into operation in 1995 with primary capacity of 10,000 m^3^/days. Two-stage treatment process was employed: primary weak water-dropping aeration (5.6 m × 3.5 m × 2.35 m, L × W × H) → primary biofiltration (6.0 m × 3.3 m × 3.75 m) → secondary mechanical surface aeration (4.5 m × 4.5 m × 4 m) → secondary biofiltration (6.0 m × 3.3 m × 3.75 m) (Additional file 1: Figure S1A). The removal of iron and manganese were ~ 90 and ~ 10% after primary biofiltration, while that increased to ~ 95% for the two contaminants after secondary biofiltration. In order to satisfy the increased water supply demand of Songbei District, the capacity increased to 40,000 m^3^/days in 2004. Meanwhile, as described in our previous study (Li et al. 2013), the theoretical oxygen demand to completely oxidize the dissolved iron, manganese and ammonia of the contaminated groundwater need to be above 7.5 mg/L, which could be calculated as: ${\text{DO}} = 0. 1 4\,C_{Fe} + 0. 2 9\,C_{Mn} + 4. 5 7\,C_{{NH_{4}^{ + } }}$ (Stumm and Morgan 2012). However, the dissolved oxygen concentration of traditional weak water-dropping aeration only reached 4.5 mg/L. Thus, we renovated the water-dropping aeration unit and combined with biofiltration process to gain synchronous removal efficiencies of the three target pollutants (Additional file 1: Figure S1B and Fig. 1a). The schematic diagram of the renovated water-dropping aeration unit was shown in Additional file 1: Figure S1C.

### Contaminated groundwater characteristics and sand filter sampling

The groundwater had been well characterized in our previous study (Li et al. 2013). As described, the dissolved iron, manganese and ammonia concentrations were 15–17, 1.5–1.7 and ~ 1.0 mg/L, which were much higher than that in the current drinking water standard (maximum permitted concentration are 0.2, 0.05 and 0.5 mg/L, respectively) (Tekerlekopoulou et al. 2013). In the biofiltration unit, the thickness of the filter media (manganese sand) is 1 m and the particle size of the filter material is 0.6–1.2 mm. The thickness of support layer is 0.75 m, of which the cobblestone block and manganese sand stone is 0.55 m (particle size of 8–32 mm) and 0.2 m (2–8 mm), respectively. The entire filter depth is 3.35 m and the depth beyond the sand surface is 2.00 m. Sand from the filter was collected for morphological observations and microbial community analysis. Sixty sand samples were taken from the upper (UL), middle (ML) and deep layers (DL) of the filter media in twenty biofilters, with the depths of about 20, 50 and 80 cm (Fig. 1a), and stored in sterilized 50 cm plastic tubes.

### DNA extraction and pyrosequencing

Prior to analysis, all the sand samples were stored at − 20 °C. PowerSoil DNA Isolation kit (Mo Bio Laboratories, Carlsbad, CA) was used to extract the genomic DNA of the sand samples according to the manufacturer’s instructions. After purification, the DNA of all the UL samples were pooled together, so as for the ML and DL samples. Polymerase chain reactions (PCRs) were performed as described previously (Sun et al. 2014). Universal primers 8F (5′-AGAGTTTGATCCTGGCTCAG-3′) and 533R (5′-TTACCGCGGCTGCTGGCAC-3′) were used to amplify V1–V3 region (length of ~ 455 bp) of the bacterial 16S rRNA gene. 454 GS-FLX sequencer was then applied for pyrosequencing according to standard protocols. Raw sequence data of the study were deposited to the NCBI Short Read Archive database with the Accession No. SRX3083403. The obtained sequences were clustered into operational taxonomic units (OTUs) using a 97 and 95% identity threshold and phylogenetically allocated down to the phylum, class and genus level with the MOTHUR program (http://www.mothur.org/wiki/Main_Page) (Wang et al. 2007). Rarefaction curves were generated and alpha diversity measurements, including the Shannon index (http://www.mothur.org/wiki/Shannon), Chao1 index (http://www.mothur.org/wiki/Chao) and ACE index (http://www.mothur.org/wiki/Ace), were calculated for each sample. CCA analysis were generated by Canoco 4.5 to evaluate the specific correlations between characteristic genera and environmental factors measured in the study, including iron, manganese, ammonia, DO, temperature, pH value and layer height. The relative abundance of 14 characteristic bacteria was used in the CCA analysis. OTUs networks were visualized in Cytoscape v3.2.1 for depicting the similarity and difference between the different microbial consortia (Zhou et al. 2017).

### Analytical methods

The water samples were filtered through a 0.45 μm cellulose nitrate membrane filter and stored at 4 °C prior to analysis. The dissolved iron and manganese concentrations were determined by atomic absorption spectrometry (Du et al. 2017). The pH value was measured by a pH meter (Mettler Toledo, Switzerland). DO was determined using a digital DO meter (HACH, USA). The ammonium concentration was measured using the APHA standard method. Morphological observations of the matured biofilter media were studied with SEM (JSM-5610). The EDS analyses were conducted immediately after the SEM images to analyze the elementary of media surface (Du et al. 2017).

## Results

### Characterization of the contaminated groundwater and filtered water

The start-up of the biofilter have been well characterized in our previous study (Li et al. 2013). We determined the iron, manganese, ammonia and DO concentrations in Songbei WTP during 30 days stable operation (Fig. 1). The dissolved iron and manganese concentrations in the contaminated groundwater were in the range of 9.4–15.7 and 1.1–1.7 mg/L. After the “aeration–biofiltration” cascading treatment, the effluent iron and manganese concentrations gradually decreased to around 0.12 and 6.0 × 10^−3^ mg/L. Meanwhile, high ammonia removal efficiency (74.1 ± 1.2%) was also achieved. Additionally, the pH and temperature were also measured, which did not change significantly under the treatment. The pH value was about 6.8. The temperature was always below 7.0 °C throughout the whole treatment, which made the treatment more difficult.

### Morphological observations of biofilter media

**Fig. 2:**
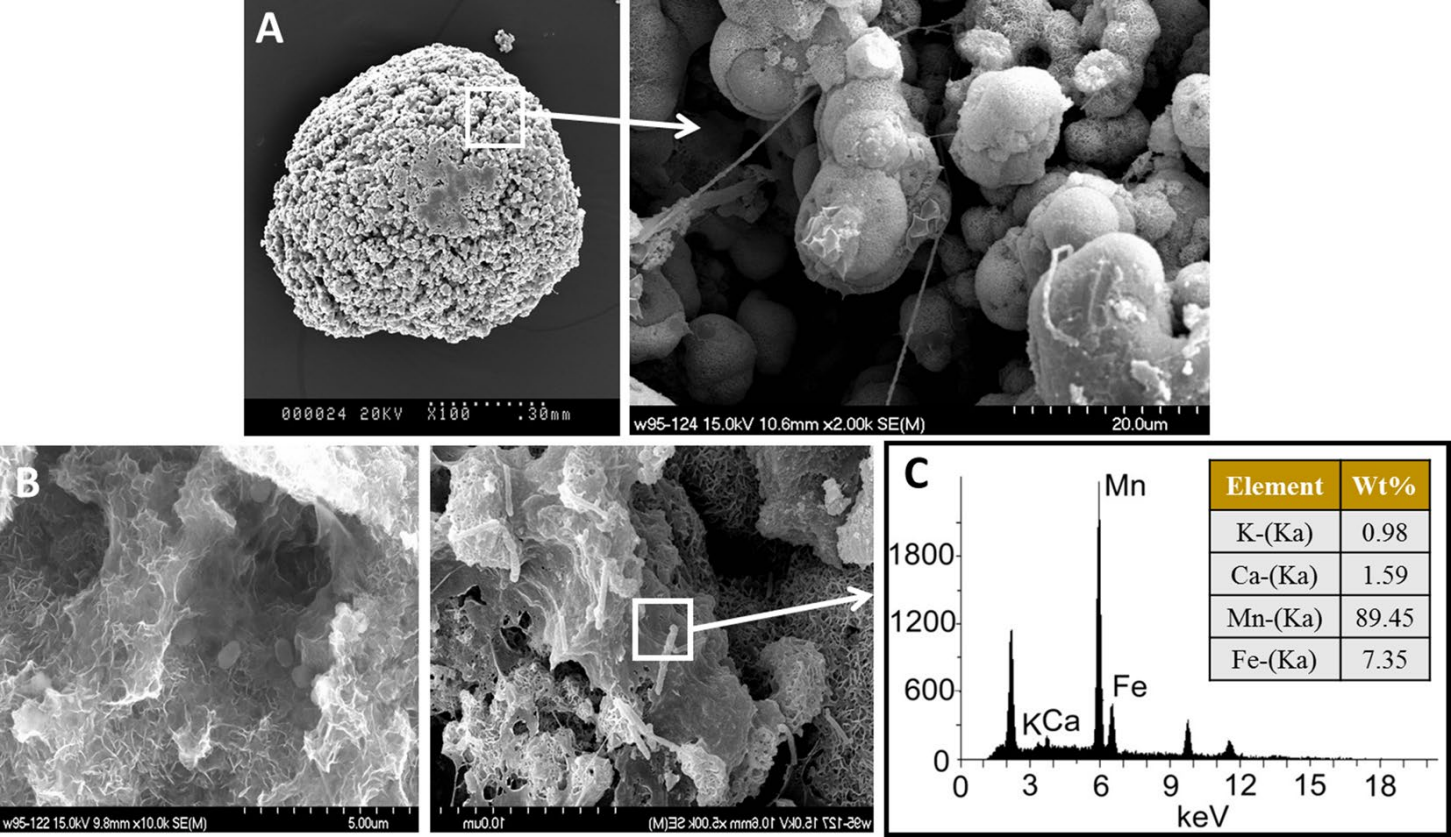
SEM images of the mature manganese sand (×100 and ×2000) (**A**). Morphology (**B**) and EDS analysis (**C**) of biofilm on the media

To have a better understanding of the biofilter media, SEM images were obtained and an EDS analysis of the manganese sand in the mature biofilter was conducted (Fig. 2). The results showed that the surface of mature media was rough and consisted of micron-sized porous particles (Fig. 2A). When magnified 2000 times, its grains (~ 20 μm) arranged loosely, which might be caused by water erosion. Furthermore, the small particles had an incomplete spherical shell membrane structure and gradually formed a sphere through layer-by-layer growth. Meanwhile, there existed a large number of microorganisms, with various morphology, and the secreted mucus [i.e., extracellular polymeric substances (EPSs)] on the biofilm of small particles (Fig. 2B). As EDS analysis of biofilm revealed (Fig. 2C), the main constituents of the mature biofilter media were manganese and iron compounds.

### Microbial community diversity

The microbial communities were characterized to compare community structure, diversity, and function in different layers. α-Diversity indexes revealed that UL biofilm sample showed the lowest diversity (Shannon index of 4.77) among three microbial communities (Additional file 1: Table S1). It was obvious that the microbial community in the upper layer of biofilter was most abundant, followed by the middle and the lower layer, regardless of the sequence divergence of 3 or 5% (Additional file 1: Figure S2A). PCoA results showed that the microbial community structure in the ML and DL samples was similar, while that in the UL sample was quite different from those two samples (Additional file 1: Figure S2B).

Phylogenetic analysis of the 16S rRNA gene sequences was performed at the phyla, class and genus level (Additional file 1: Figure S3). The two dominant phyla for UL, ML and DL samples were the same but with different relative abundance, which were *Proteobacteria* with 40.4–56.1%, followed by *Nitrospirae* with 11.6–23.9% in the individual samples (Additional file 1: Figure S3A). *Proteobacteria* decreased with biofilter depths, while *Nitrospirae* peaked at the ML sample. The dominant class in the sand filter was the *Betaproteobacteria*, *Nitrospira*, *Alphaproteobacteria* and *Actinobacteria* (Additional file 1: Figure S3B). These class were found in different amounts in the three samples, which is in accordance with other studies of freshwater sediments (Nitzsche et al. 2015; White et al. 2012). Their relative abundance together accounted for 70.7% of all bacterial 16S rRNA gene sequences in the UL sample, and 57.2 and 48.4% in the ML and DL samples, respectively.

The relative abundance of bacteria at the genus level is shown in Additional file 1: Figure S3C. *Propionibacterium* took up the largest proportion (17.5%) in the UL sample, which have been shown to be a nitrate-reducing bacteria (NRB) and capable of producing N_2_O as an end product (Kaspar et al. 1981). Interestingly, this genera were not detected in the ML and DL samples. *Nitrosomonas* (8.9%), which are known to be ammonia oxidizers (AOB), also clearly dominated in the UL community. Previous research demonstrated that *Nitrosomonas* are better adapted to low ammonia concentrations than other AOBs (Regan et al. 2003; Štembal et al. 2005). *Candidatus Nitrotoga*, as cold-adapted nitrite-oxidizing bacteria (NOB) previously isolated from activated sludge (Alawi et al. 2007), peaked at 3.5% in the UL sample. This may give beneficial operational flexibility to the groundwater biofilter due to these organisms could oxidize nitrite at temperatures near 4 °C (Alawi et al. 2007). *Nitrospira* (23.9%) dominated in the ML sample, which is expected to be the dominant NOBs due to their lower half-saturation coefficient for oxygen and nitrite (Manser et al. 2005). *Delftia* (6.6%), *Gemmatimonas* (3.4%) and *Haliscomenobacter* (3.1%) dominated in the DL sample. These results showed that there were significant differences among microbial community compositions in different layers of the biofilter.

## Discussion

### Overall performance of Songbei WTP

After the “aeration–biofiltration” cascading treatment, the effluent iron, manganese and ammonia were all far below the permitted concentration level in the current drinking water standard. Figure 1e showed that the DO achieved 8.5 mg/L in the renovated aeration unit, which totally meet the demand of iron, manganese and ammonia oxidization in the raw groundwater. The DO concentration decreased to ~ 1.5 mg/L in the effluent of biofilter. The results of SEM and EDS analysis showed that the manganese and iron compounds attached on the surface of mature biofilter media, which could be the direct evidence for the oxidation of iron and manganese in the biofilter media by microbial catalysis. This result was also in accordance with previous studies (Du et al. 2017; Li et al. 2013). Simultaneously, the EPSs secreted by microorganisms and the sheet membrane structure composed of iron and manganese oxides provided the living space for the growth of microorganisms. With the further development of biofilms and oxides deposition, the incomplete spherical shell could gradually form to small manganese particles.

### Functional bacteria distribution

**Fig. 3:**
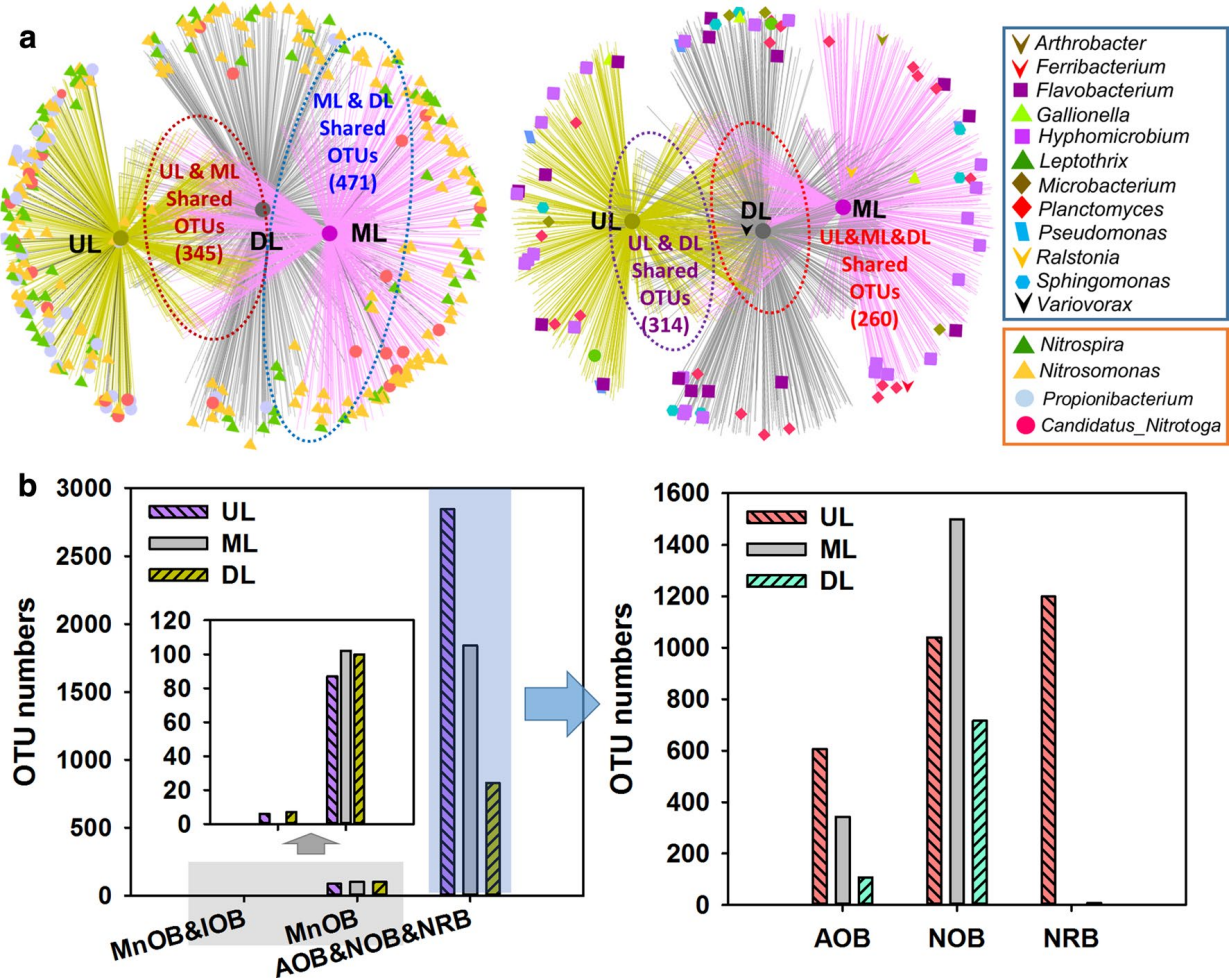
OTU networks of specific functional genera in the three bacterial communities (**a**). Genus level identification of the specific functional bacterial sequences (Unit: Number of OTUs) (**b**)

As previously noted, the biological process played an important role in the biofilters for the removal of the three pollutants from groundwater. To elucidate the interactions among all of the OTUs and analyze the shared and most abundant OTUs in the three microbial consortia samples, a network representing the functional bacteria community change and linkage was constructed (Fig. 3a). Collectively, only 260 phylum-level OTUs, out of 2478 in total, were shared in these three samples. The number of OTUs shared by the UL and ML samples was 345, while 314 and 471 were shared by the UL&DL and ML&DL samples, respectively (Fig. 3a). This result was accordance with the PCoA analysis. The majority of the shared OTUs were *Proteobacteria* (44.2–53.0%), *Acidobacteria* (9.2–11.0%) and *Nitrospirae* (7.3–8.5%). Nitzsche et al. (2015) showed that the sand filter community was to a large extent dominated by nitrifying bacteria (Nitzsche et al. 2015). In this study, the bacteria capable of ammoxidation and nitrification, i.e., *Propionibacterium*, *Nitrosomonas*, *Nitrosomonas* and *Candidatus Nitrotoga*, accounted for 41.6% (8.9% AOB, 15.2% NOB and 17.5% NRB) of the population in the UL sample, which was 1.3 and 2.2-fold of that in the ML and DL samples (Fig. 3b). Further study on the specific composition of nitrifying bacteria showed that the NRB were dominated in the UL sample (17.5%), while NOB was in the ML sample (26.1%). Compared with the ammonia-oxidizing and nitrifying bacteria, only low numbers of OTUs related to known iron- and manganese-oxidizing bacteria (IOB and MnOB) in the three sand filter samples were quantified (< 0.2%, data not shown). Especially for the IOB, i.e., *Gallionella* and *Leptothrix* (Yang et al. 2014), there were only 6 and 7 OTUs in the UL and DL samples. Such high iron removal efficiency maybe caused by the synergistic effects of oxygen auto-catalytic and biological iron oxidation, which was in accordance with the findings of Voegelin et al. (2014) and Yang et al. (2014). By contrast, a wide range of MnOB bacterial genera was identified, including *Flavobacterium* (Cai et al. 2015), *Hyphomicrobium* (Yang et al. 2014), *Planctomyces* (Yang et al. 2014), *Ralstonia* (Yang et al. 2013) and *Sphingomonas* (Li et al. 2016). However, only a low relative sequence abundance of OTUs were found (Fig. 3a), the no. of all the related MnOB peaked at 102 in the ML sample, followed by the DL sample (100). The reason behind here maybe that the biological Mn oxidation was induced by microbial consortia involved in this study. Nitzsche et al. (2015) also found that only low OTUs numbers related to known IOB and none of MOB were identified in a household sand filter. They suggested that biotic Mn oxidation was most likely mediated by a phylogenetically diverse microbial community (Nitzsche et al. 2015). It is well known that the sites for the dissolved iron, manganese and ammonia removal were separated orderly along the depth of the biofilter. That is, most of the iron and ammonia were removed in the upper layer of biofilter and manganese removal was mainly concentrated in the lower layer. This study confirm the unanimous results of previous research (Li et al. 2013; Nitzsche et al. 2015; Tekerlekopoulou et al. 2013).

### Correlation analysis between functional bacteria and environmental variables

**Fig. 4:**
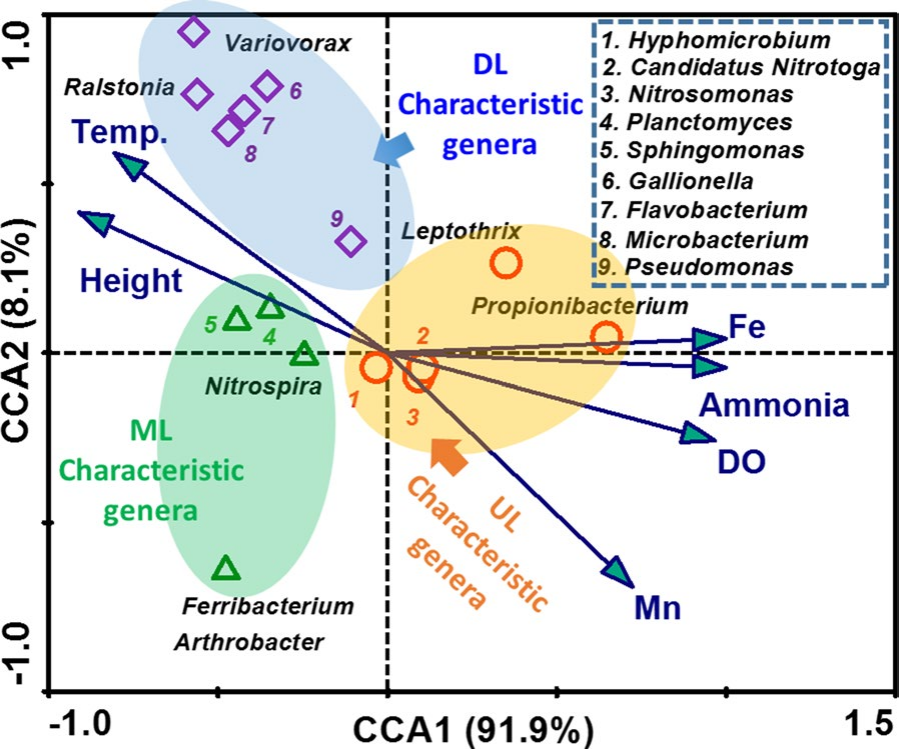
Canonical correspondence analysis (CCA) between enriched genera and environmental variables (iron, manganese, ammonia, DO, temperature and layer height)

To further discern the plausible correlation between biofilter samples, characteristic genera and various environmental and performance measurements, CCA analysis was performed in this study (Fig. 4). 16 characteristic bacteria and 6 environmental variables were taken into consideration. The CCA1 and CCA2 model explained 91.9 and 8.1% of total variance, which indicate that these two coordinates could represent the CCA results. The dissolved iron, manganese, ammonia and DO concentrations were positively correlated with the first canonical axis. For axis 2, only dissolved iron, layer height and temperature showed good positive correlations. The detailed information is shown in Additional file 1: Table S2. According to the length of the vector, indicating the strength of the relationship between the environmental variable and microbial community, all the six variables strongly linked to the microbial community. The characteristic genera in the UL sample had a highly positive correlation with the iron and ammonia removal. It is worthwhile to note that *Propionibacterium* was comparatively correlated with NH_4_^+^ removal. We also found that Mn removal had very high positive correlation with a number of functional bacteria, such as *Ralstonia*, *Variovorax*, *Gallionella*, *Flavobacterium*, *Microbacterium*, which were abundant in the DL sample. That is, the removal of iron and ammonia was prior to that of manganese, which occurred at the upper and deeper layers of biofilter, respectively. This was consistent with the above discussions (Functional bacteria distribution). In addition, the intersection angle between the iron and DO was slightly greater than that of ammonia, indicating that the ammonia removal was more related to DO concentration than iron. This could be verified by the coefficients of DO demand for the iron and ammonia removal, which were 0.14 and 4.57, respectively (Stumm and Morgan 2012). In this sense, the CCA results suggested that the stable biofilm on the biofilter media, created by certain microorganisms from the groundwater microflora, may play a crucial role in the simultaneous removal of iron, ammonia and manganese. Meanwhile, the relationship between the functional bacteria and the environmental variables may provide insight on possible mechanism of the “aeration–biofiltration” cascading treatment process.

## Additional file

**Additional file 1.** Additional figures and tables.

## References

[CR1] Alawi M, Lipski A, Sanders T, Pfeiffer EM, Spieck E (2007). Cultivation of a novel cold-adapted nitrite oxidizing betaproteobacterium from the Siberian Arctic. ISME J Multidiscip J Microb Ecol.

[CR2] Cai Y, Li D, Liang Y, Luo Y, Zeng H, Zhang J (2015). Effective start-up biofiltration method for Fe, Mn, and ammonia removal and bacterial community analysis. Bioresour Technol.

[CR3] Du X, Liu G, Qu F, Li K, Shao S, Li G, Liang H (2017). Removal of iron, manganese and ammonia from groundwater using a PAC-MBR system: the anti-pollution ability, microbial population and membrane fouling. Desalination.

[CR4] Hasan HA, Abdullah SRS, Kamarudin SK, Kofli NT (2013). On–off control of aeration time in the simultaneous removal of ammonia and manganese using a biological aerated filter system. Process Saf Environ Prot.

[CR5] Kaspar HF, Tiedje JM, Firestone RB (1981). Denitrification and dissimilatory nitrate reduction to ammonium in digested sludge. Can J Microbiol.

[CR6] Li XK, Chu ZR, Liu YJ, Zhu MT, Yang L, Zhang J (2013). Molecular characterization of microbial populations in full-scale biofilters treating iron, manganese and ammonia containing groundwater in Harbin, China. Bioresour Technol.

[CR7] Li C, Wang S, Du X, Cheng X, Fu M, Hou N, Li D (2016). Immobilization of iron- and manganese-oxidizing bacteria with a biofilm-forming bacterium for the effective removal of iron and manganese from groundwater. Bioresour Technol.

[CR8] Manser R, Gujer W, Siegrist H (2005). Consequences of mass transfer effects on the kinetics of nitrifiers. Water Res.

[CR9] Nitzsche KS, Weigold P, Lösekann-Behrens T, Kappler A, Behrens S (2015). Microbial community composition of a household sand filter used for arsenic, iron, and manganese removal from groundwater in Vietnam. Chemosphere.

[CR10] Pacini VA, Ingallinella AM, Sanguinetti G (2005). Removal of iron and manganese using biological roughing up flow filtration technology. Water Res.

[CR11] Regan JM, Harrington GW, Baribeau H, De Leon R, Noguera DR (2003). Diversity of nitrifying bacteria in full-scale chloraminated distribution systems. Water Res.

[CR12] Štembal T, Markić M, Ribičić N, Briški F, Sipos L (2005). Removal of ammonia, iron and manganese from groundwaters of northern Croatia—pilot plant studies. Process Biochem.

[CR13] Stumm W, Morgan JJ (2012). Aquatic chemistry: chemical equilibria and rates in natural waters.

[CR14] Sun R, Zhou A, Jia J, Liang Q, Liu Q, Xing D, Ren N (2014). Characterization of methane production and microbial community shifts during waste activated sludge degradation in microbial electrolysis cells. Bioresour Technol.

[CR15] Tang J, Bian J, Li Z, Li Y, Yang W, Liang S (2016). Comparative study on the hydrogeochemical environment at the major drinking water based arsenism areas. Appl Geochem.

[CR16] Tekerlekopoulou AG, Pavlou S, Vayenas DV (2013). Removal of ammonium, iron and manganese from potable water in biofiltration units: a review. J Chem Technol Biotechnol.

[CR17] Voegelin A, Kaegi R, Berg M, Nitzsche KS, Kappler A, Lan VM, Trang PTK, Göttlicher J, Steininger R (2014). Solid-phase characterisation of an effective household sand filter for As, Fe and Mn removal from groundwater in Vietnam. Environ Chem.

[CR18] Wang Q, Garrity GM, Tiedje JM, Cole JR (2007). Naïve Bayesian classifier for rapid assignment of rRNA sequences into the new bacterial taxonomy. Appl Environ Microbiol.

[CR19] White CP, Debry RW, Lytle DA (2012). Microbial survey of a full-scale, biologically active filter for treatment of drinking water. Appl Environ Microbiol.

[CR20] Yang W, Zhang Z, Zhang Z, Chen H, Liu J, Ali M, Liu F, Li L (2013). Population structure of manganese-oxidizing bacteria in stratified soils and properties of manganese oxide aggregates under manganese-complex medium enrichment. PLoS ONE.

[CR21] Yang L, Li X, Chu Z, Ren Y, Zhang J (2014). Distribution and genetic diversity of the microorganisms in the biofilter for the simultaneous removal of arsenic, iron and manganese from simulated groundwater. Bioresour Technol.

[CR22] Zhou A, Zhang J, Varrone C, Wen K, Wang G, Liu W, Wang A, Yue X (2017). Process assessment associated to microbial community response provides insight on possible mechanism of waste activated sludge digestion under typical chemical pretreatments. Energy.

