# Supplementary material for: Microbial community response reveals underlying mechanism of industrial-scale manganese sand biofilters used for the simultaneous removal of iron, manganese and ammonia from groundwater
Source: AMB Express. 2018 Jan 8;8:2. doi: 10.1186/s13568-017-0534-7 (PMC5758488; doi:10.1186/s13568-017-0534-7)
Supplement: Supplementary file 1 — Additional file 1. Additional figures and tables. [file 13568_2017_534_MOESM1_ESM.pdf]

Additional file 1

Microbial community response reveals underlying mechanism of industrial-scale manganese sand biofilters used for the simultaneous removal of iron, manganese and ammonia from groundwater

Yu Zhang, Rui Sun [*], Aijuan Zhou, Jiaguang Zhang, Jianna Jia, Yanan Li, Xiuping Yue, Jie Zhang

***Corresponding author.** E-mail: 05doudou@163.com (R. Sun)

Contents

Additional tables (Table S1 and S2)

Additional figures (Figures S1, S2 and S3)

Additional tables

Table S1. Alpha diversity of three bacterial samples

Name*1	Seq Num*[1]	Barcoded primers	Shannon index	ACE index	Chao 1 index	Coverage
UL	6842	AGCTAGATAC	4.77	1610	1167	0.95
ML	5739	AGCTGTCGAC	5.56	2029	1549	0.93
DL	4428	AGTATGCACG	5.66	1819	1405	0.91

*[1]: “Seq num” indicated the sequence numbers obtained from the high-throughput sequencing analysis;

Table S2. The eigenvalues of first two canonical axes and their relationships with each environmental factor

	Axis 1	Axis 2
Eigenvalues	0.338	0.030
Cumulative percentage variance	91.9	100.0
Ammonia	0.9990	-0.0439
Mn	0.7238	-0.6900
Fe	0.9991	0.0429
DO	0.9662	-0.2576
Temperature	-0.8057	0.5923
Layer height	-0.9096	0.4156

Additional figures

Figure captions

Figure S1 The schematic diagrams of traditional two-stage treatment process (A), the enhanced one-stage treatment process (B) and the renovated water-dropping aeration unit (C).

Figure S2 Rarefaction curves (A) and principal coordinate analysis (PCoA) (B) of bacterial communities from three biofilter samples based on pyrosequencing of 16S rRNA gene. PCoA1 and PCoA2 axes represent 61.87% and 32.5% of the variance within the microbial community.

Figure S3 Taxonomic classification of pyrosequences from the three bacterial communities at the phylum (a), class (b) and genus (c) levels.

Figure S1

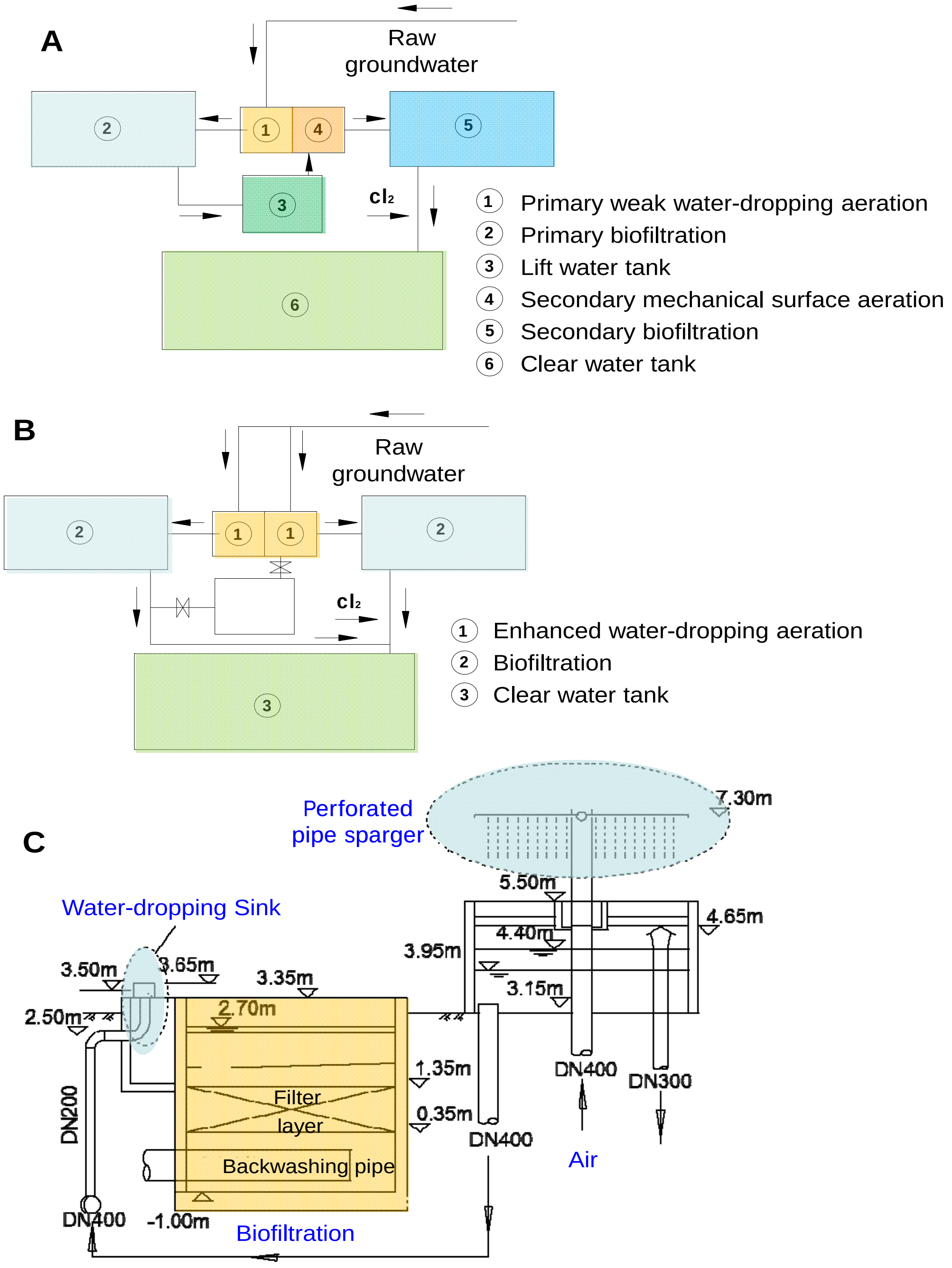
A
Raw groundwater
Cl2
1 Primary weak water-dropping aeration
2 Primary biofiltration
3 Lift water tank
4 Secondary mechanical surface aeration
5 Secondary biofiltration
6 Clear water tank
B
Raw groundwater
Cl2
1 Enhanced water-dropping aeration
2 Biofiltration
3 Clear water tank
C
Perforated pipe sparger
7.30m
5.50m
4.65m
4.40m
3.95m
3.15m
Water-dropping Sink
3.50m
3.65m
3.35m
2.50m
2.70m
Filter layer
Backwashing pipe
1.35m
0.35m
DN200
DN400
DN400
DN400
DN300
Air
-1.00m
Biofiltration

Figure S2

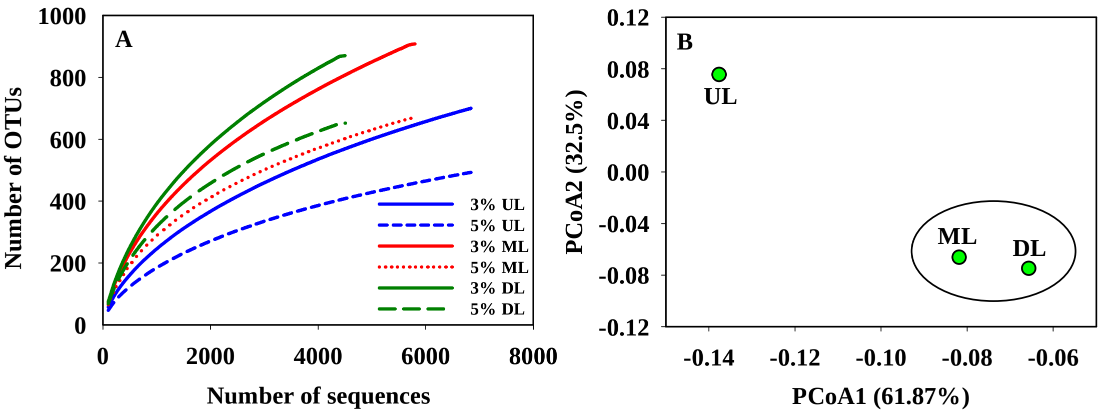
A
Number of OTUs
Number of sequences
3% UL
5% UL
3% ML
5% ML
3% DL
5% DL
B
UL
ML
DL
PCoA2 (32.5%)
PCoA1 (61.87%)

Figure S3

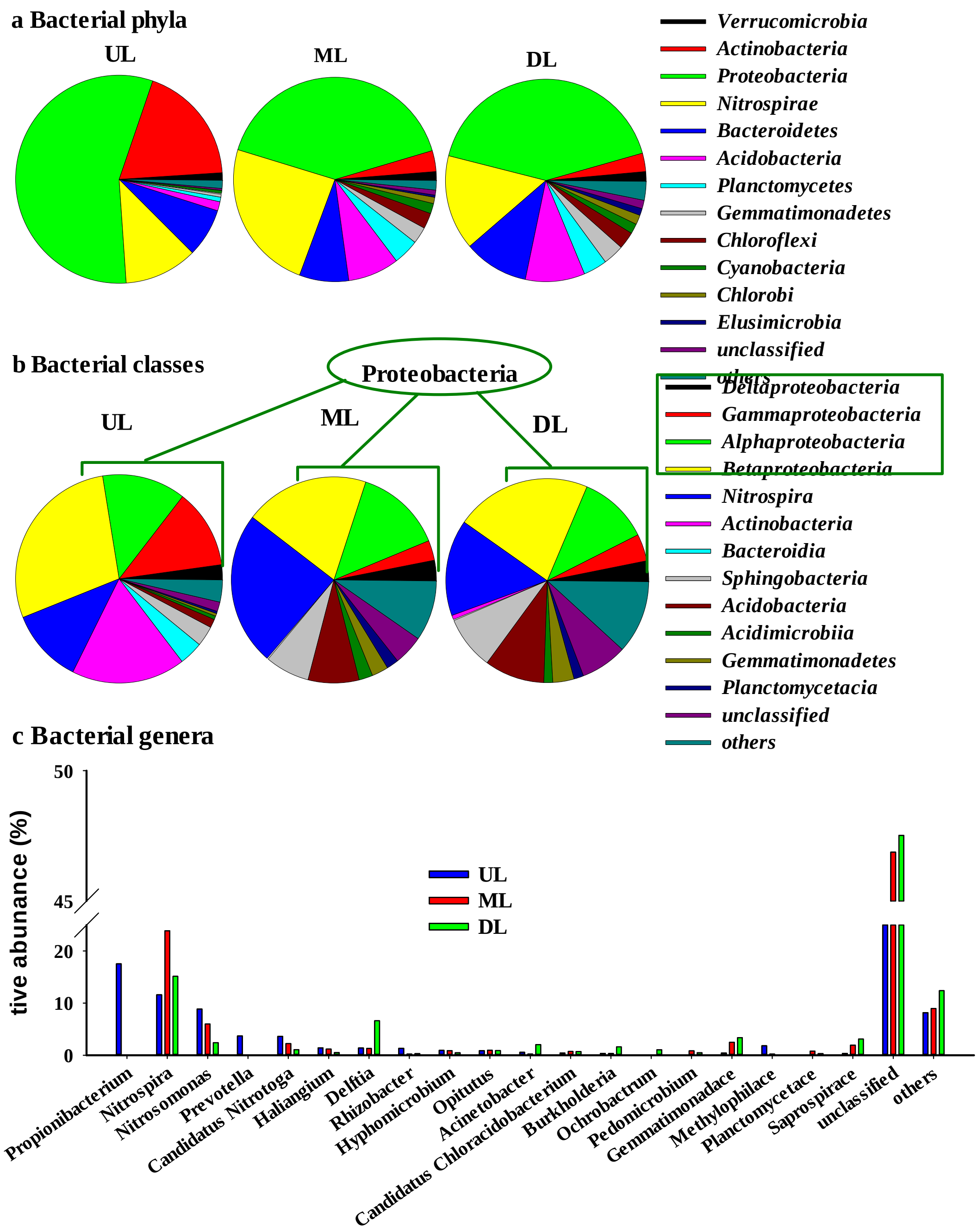

a Bacterial phyla
UL
ML
DL
Verrucomicrobia
Actinobacteria
Proteobacteria
Nitrospirae
Bacteroidetes
Acidobacteria
Planctomycetes
Gemmatimonadetes
Chloroflexi
Cyanobacteria
Chlorobi
Elusimicrobia
unclassified
others
b Bacterial classes
Proteobacteria
UL
ML
DL
Deltaproteobacteria
Gammaproteobacteria
Alphaproteobacteria
Betaproteobacteria
Nitrospira
Actinobacteria
Bacteroidia
Sphingobacteria
Acidobacteria
Acidimicrobiia
Gemmatimonadetes
Planctomycetacia
unclassified
others
c Bacterial genera
tive abunance (%)
50
45
20
10
0
UL
ML
DL
Propionibacterium
Nitrospira
Nitrosomonas
Prevotella
Candidatus Nitrotoga
Haliangium
Delftia
Rhizobacter
Hyphomicrobium
Opitutus
Acinetobacter
Candidatus Chloracidobacterium
Burkholderia
Ochrobactrum
Pedomicrobium
Gemmatimonadace
Methylophilace
Planctomycetace
Saprospirace
unclassified
others
